# Non-steroidal Anti-inflammatory Drug-Induced Severe Eosinophilia and Hematogone Hyperplasia Masquerading as Leukemia: Diagnostic Complexity in a Young Child

**DOI:** 10.7759/cureus.99379

**Published:** 2025-12-16

**Authors:** Shruti Saxena, Savitri Singh, Nita Radhakrishnan

**Affiliations:** 1 Department of Pediatric Hematology Oncology, Post Graduate Institute of Child Health, Noida, IND; 2 Department of Pathology, Post Graduate Institute of Child Health, Noida, IND

**Keywords:** drug-induced severe eosinophilia, eosinophilia in pediatric patients, hematogone hyperplasia, hematogone hyperplasia masquerading leukemia, nsaid-induced severe eosinophilia

## Abstract

Eosinophilia in pediatric patients may arise from a wide spectrum of underlying etiologies encompassing reactive, clonal, and idiopathic disorders. While reactive eosinophilia is common in childhood and typically related to infections, allergic or atopic disease, hypersensitivity reactions, or systemic inflammatory disorders, clonal eosinophilia is uncommon and may be associated with primary hematological malignancies. The distinction between reactive and clonal eosinophilia is clinically crucial, particularly in the presence of alarming features such as extreme leukocytosis, organomegaly, cytopenias, or circulating blasts that may mimic acute leukemia. We present the case of a three-year-old boy with severe eosinophilia and anemia following a short febrile illness and exposure to a non-steroidal anti-inflammatory drug combination, raising an initial suspicion of leukemia. Bone marrow examination and flow cytometry revealed significant hematogone hyperplasia with no evidence of acute leukemia, supporting a diagnosis of drug-induced reactive eosinophilia. The coexistence of hematogone hyperplasia and eosinophilia in this clinical setting has not been previously described. This case emphasizes the importance of systematic evaluation to avoid misdiagnosis and unnecessary evaluation and treatment.

## Introduction

Eosinophilia in childhood is defined as an elevated absolute eosinophil count (AEC) above the age-adjusted physiological upper limit, which is typically between 350 and 500 cells/µL in healthy children. Eosinophilia may be categorized based on severity as mild (500-1,500 cells/µL), moderate (1,500-5,000 cells/µL), or severe (>5,000 cells/µL), and can be reactive, clonal, or idiopathic. Reactive eosinophilia represents a polyclonal increase in mature eosinophils driven by cytokines such as interleukin (IL)-3, IL-5, and granulocyte-macrophage colony-stimulating factor produced in response to immunological stimulation. In contrast, clonal eosinophilia results from expansion of neoplastic hematopoietic precursors and is typically associated with myeloproliferative neoplasms or acute leukemia [1-3].

In children, reactive eosinophilia is far more common than clonal disease, with frequent associations including parasitic infections, allergic disorders, autoimmune disease, drug hypersensitivity, inflammatory bowel disease, and malignancies, especially acute lymphoblastic leukaemia and Hodgkin lymphoma [1]. Severe eosinophilia accompanied by cytopenias, systemic symptoms, or morphological atypia warrants comprehensive evaluation to rule out underlying malignancy or primary hypereosinophilic syndromes.

Primary eosinophilic disorders are rare in the pediatric population and include chronic eosinophilic leukemia; myeloid/lymphoid neoplasms with rearrangements of *PDGFRA*, *PDGFRB*, *FGFR1*, or *PCM1-JAK2*; and idiopathic hypereosinophilic syndrome [2]. These disorders present diagnostic difficulty due to significant clinical overlap with reactive eosinophilia.

Bone marrow examination plays a pivotal role in evaluating unexplained severe eosinophilia, particularly when the peripheral smear demonstrates abnormal morphology or circulating blasts. Flow cytometry is essential to differentiate benign precursor lymphoid proliferation (hematogone hyperplasia) from malignant lymphoblast populations. Hematogones are benign immature B-cell precursors normally identified in the bone marrow of infants and young children, typically decreasing with age [4]. While increased hematogones are frequently noted following bone marrow regeneration, chemotherapy, transplantation, or immune-mediated cytopenias, their association with drug-induced severe eosinophilia has not been previously documented.

We describe a case of severe eosinophilia following analgesic exposure and angioedema, presenting with morphological abnormalities concerning for acute leukemia, but ultimately diagnosed as drug-induced eosinophilia with hematogone hyperplasia. This case highlights essential diagnostic considerations in pediatric eosinophilia.

## Case presentation

A 3-year-old male child, previously healthy and developmentally appropriate, born to non-consanguineous parents, presented with a two-day history of fever followed by sudden-onset generalized edema. He had no prior history of atopy, asthma, autoimmune disease, chronic infection, parasitic infestation, or similar episodes. There was no family history of hematologic or allergic disorders.

During the febrile episode, he received a commercially available antipyretic preparation containing aceclofenac and paracetamol at recommended dosing. Approximately 24 hours after commencing the medication, he developed progressive edema involving the periorbital tissues, lips, and scrotum. He was evaluated at a local hospital, where treatment with pheniramine maleate and hydrocortisone led to partial symptomatic improvement. Initial hematological investigations revealed significant leukocytosis, prompting further evaluation. He had no rash, wheezing, joint symptoms, vomiting, diarrhea, or abdominal pain.

Routine laboratory tests showed marked eosinophilia, with serial hematological indices demonstrating a progressive rise in leukocyte count and AEC (Table 1).

**Table 1:** Serial hemoglobin, white blood cell, eosinophil, and platelet counts of the patient. Hb = hemoglobin; TLC = total leukocyte count; DIC = differential leukocyte count; AEC = absolute eosinophil count

	Normal	14/10/21	17/10/21	23/10/21	01/11/21	15/11/21	20/11/21	03/12/21	05/01/22
Hb (g/dL)	11.5–13.5	8.2	7.5	-	-	8.74	7.96	8.1	9.4
TLC (cells/mL)	5,500–15,500	24,700	36,800	43,269	47,000	37,400	36,700	11,200	8,200
DLC	-	N_68_L_28_E_4_	N_58_L_30_E_12_	N_14_L_34_E_52_	E_40_	N_12_L_11_E_70_	N_10_L_26_E_58_	N_42_L_51_E_0.1_	N_56_L_34_E_1_
AEC (cells/mL)	165–465	988	4,416	22,500	18,960	26,400	21,285	926	-
Platelet (cells/mL)	200,000–450,000	346,000	308,000	-	-	365,000	432,000	221,000	212,000

Urine testing revealed proteinuria (3+), and serum IgE was markedly elevated (9,773 kAU/L; normal <32 kAU/L). A provisional diagnosis of allergic interstitial nephritis secondary to drug hypersensitivity was considered. However, the persistence of severe eosinophilia along with anemia led to referral for suspected acute leukemia.

At presentation to our center, the child appeared clinically stable, afebrile, and interactive. He was pale, with persistent mild periorbital edema, but showed no icterus, petechiae, lymphadenopathy, hepatomegaly, or splenomegaly. Cardiovascular and respiratory examinations were unremarkable.

Laboratory evaluation

**Figure 1:**
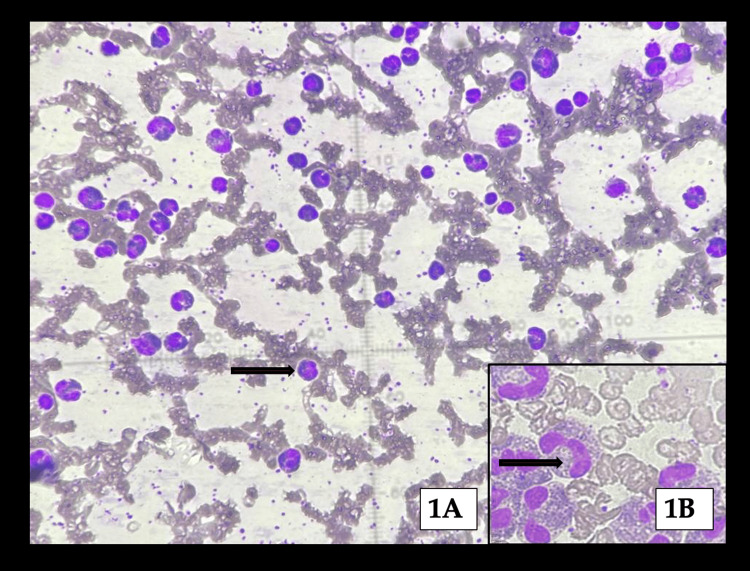
Low-power view of hypercellular bone marrow with dense eosinophilic proliferation (black arrow) (A), and high-power inset highlighting atypical eosinophilic precursors/blasts (black arrow) (B).

A complete blood count demonstrated persistent leukocytosis with an AEC exceeding 26,000/µL at peak. Peripheral blood smear showed microcytic hypochromic anemia, target cells, poikilocytosis, myelo-eosinophils, and large granular lymphocytes. Eosinophils displayed atypical features, including hypergranulation, coarse granules, and cytoplasmic vacuolization. Circulating atypical lymphoid cells prompted concern for lymphoid malignancy. Bone marrow aspiration revealed hypercellularity with marked myeloid hyperplasia and dysplastic eosinophils (Figure 1).

Plasma cells were increased, and 7% atypical lymphoid cells were present; megakaryocytes demonstrated mild dysplasia. Figure 1 demonstrates bone marrow aspirate with trilineage hematopoiesis and markedly increased cellularity (~90%). Myeloid series was predominantly eosinophilic, including myelocytes and eosinophils, admixed with scattered mononuclear cells with a high nuclear-to-cytoplasmic ratio and occasional atypical cells (~4%). Megakaryocytes were present in paratrabecular and interstitial regions, with patchy eosinophilic material deposition.

**Figure 2:**
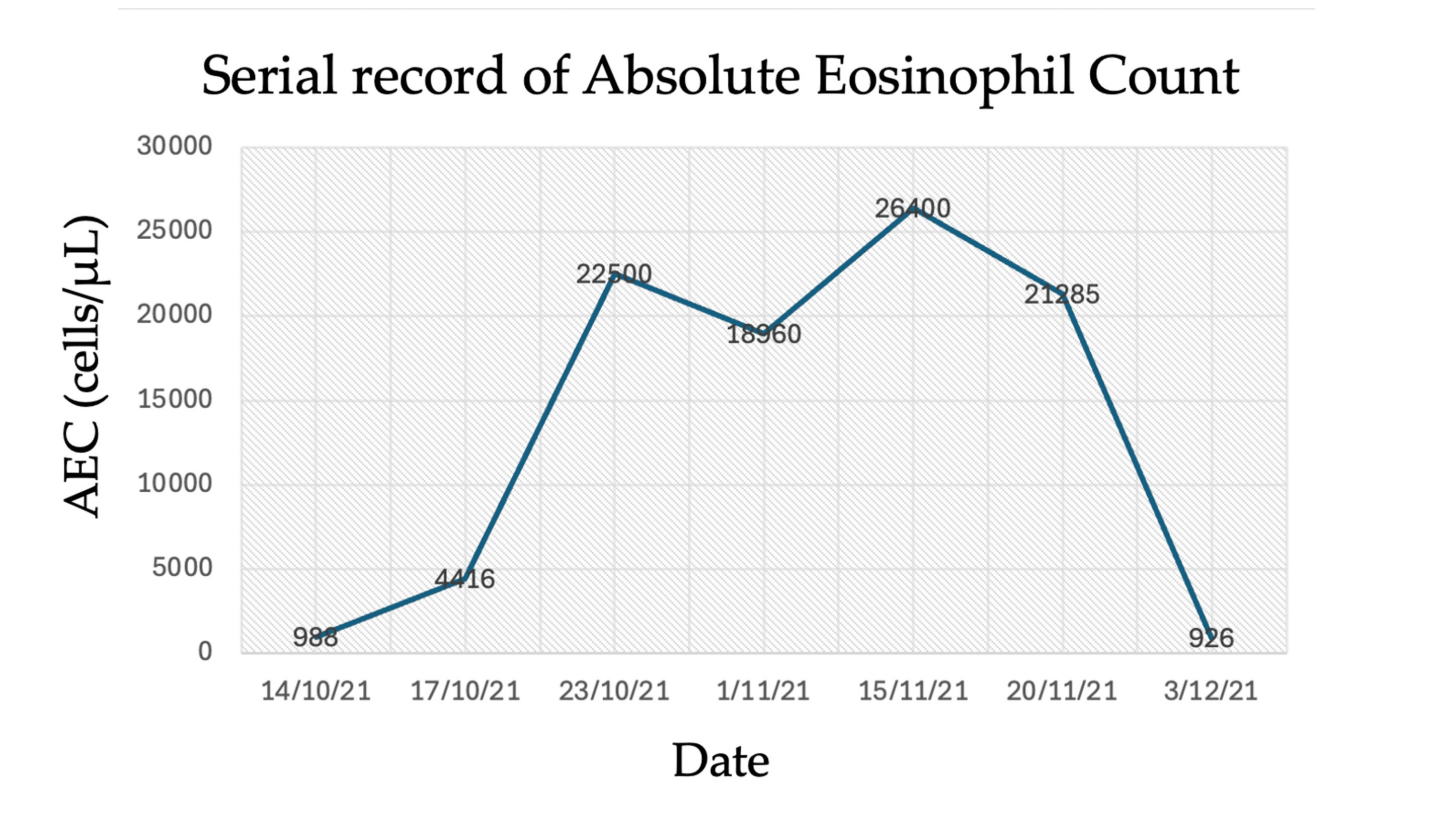
Trend of absolute eosinophil count (AEC) demonstrating marked eosinophilia with peak values exceeding 26 × 10³/µL, followed by resolution to near-normal levels over sequential measurements.

Figure 2 demonstrates a trend of AEC demonstrating marked eosinophilia with peak values exceeding 26 × 10³/µL, followed by resolution to near-normal levels over sequential measurements.

In view of atypical eosinophils, flow cytometric evaluation of the bone marrow sample was performed using a stain-lyse-wash preparation on a BD FACS Canto platform with BD FACS DIVA software, applying SSC versus CD45 gating to interrogate all leukocyte populations. The comprehensive acute leukemia diagnostic panel included stem and progenitor markers (CD34, CD117), immaturity markers (TdT, HLA-DR), and lineage-associated markers for myeloid, B- and T-lymphoid, natural killer (NK), and monocytic differentiation. The analysis demonstrated 0.8% myeloid blasts and a markedly increased population of B-cell precursors (hematogones; 10.7%), alongside mature T-lymphocytes (9.5%), mature B-lymphocytes (4.5%), NK cells (3.5%), monocytes (2.9%), and maturing myeloid elements with prominent eosinophils, with immunophenotypic findings showing no aberrant expression suggestive of neoplasia. The overall profile was interpreted as hematogone hyperplasia without evidence of acute leukemia, a reactive regenerative pattern observed in association with marrow recovery following cytopenias, viral infections, solid tumors, or post-myelosuppressive therapy and transplantation.

Clinical course

Iron supplementation (3 mg/kg/day) was initiated for iron deficiency anemia. As eosinophil counts demonstrated spontaneous improvement, no corticosteroids or cytotoxic therapy were administered. The AEC normalized over 14 days, concurrent with rising hemoglobin levels. The family was counseled to avoid non-steroidal anti-inflammatory drugs (NSAIDs) in the future due to suspected drug-induced hypersensitivity.

## Discussion

Severe eosinophilia in a child, particularly accompanied by anemia and peripheral atypical cells, often raises concern for hematological malignancy. However, reactive eosinophilia remains the most frequent cause in pediatric practice. The mechanism of reactive eosinophilia in hypersensitivity reactions is driven by IL-5-mediated eosinophilopoiesis, mast cell activation, and IgE-linked Th2 inflammation. This case demonstrated a strong temporal correlation between drug exposure, angioedema, elevated IgE, and eosinophilia, supporting a diagnosis of drug-induced eosinophilia.

NSAIDs are well-recognized triggers of hypersensitivity reactions, although aceclofenac-induced eosinophilia is rarely reported in the literature. The presence of proteinuria and edema suggests transient renal involvement consistent with allergic interstitial nephritis. Additionally, the transient elevation of atypical lymphoid cells is consistent with reactive immunological stimulation.

Hematogone hyperplasia and diagnostic confusion

Hematogones are normal B-lymphocyte precursors in bone marrow that under physiologic conditions constitute only a small fraction of marrow cellularity [4]. Their expansion beyond approximately 5% constitutes hematogone hyperplasia, often occurring in reactive settings such as marrow regeneration after cytotoxic injury, infections, autoimmune cytopenias, or after bone marrow transplantation. Increased hematogones must be differentiated from precursor B-lymphoblasts seen in acute lymphoblastic leukemia. Flow cytometry is indispensable, as reliance on morphology alone may result in misdiagnosis.

Hematogone hyperplasia is typically encountered in clinical contexts characterised by marrow regeneration or immune dysregulation. It frequently emerges during recovery from bone marrow suppression following chemotherapy or hematopoietic stem cell transplantation, and may also be identified in the setting of severe systemic infections or immune-mediated cytopenias. Nutritional deficiencies such as vitamin B12 or copper deficiency have been associated with marked expansion of hematogones, and similar findings are reported in autoimmune disorders and congenital bone marrow failure syndromes. Viral infections, particularly those due to cytomegalovirus and Epstein-Barr virus, are well-recognized triggers of hematogone proliferation, reflecting reactive lymphoid regeneration rather than clonal neoplasia [4-6].

By contrast, the published data on NSAIDs exerting a stimulatory effect on marrow lymphoid precursor cells is very limited. Some experimental work suggests that certain non-selective cyclooxygenase inhibitors may stimulate the proliferation of hematopoietic stem cells in murine models and thereby enhance marrow and splenic hematopoiesis [7]. However, other in vitro studies on human bone marrow mesenchymal or stromal cells exposed to NSAIDs report suppression of proliferation, cell-cycle arrest, or impaired osteoblastic differentiation rather than lymphoid expansion [8].

Severe pediatric eosinophilia encompasses a broad differential that includes reactive, clonal, and idiopathic causes. Reactive eosinophilia is most frequently triggered by infections, including parasitic, protozoal, bacterial, fungal, and viral pathogens, as well as allergic disorders, drug hypersensitivity, autoimmune diseases such as sarcoidosis, inflammatory bowel disease, and autoimmune lymphoproliferative syndrome. Additional contributors include miscellaneous systemic conditions such as hypoadrenalism and radiation exposure, along with neoplastic disorders, including leukemia, lymphoma, solid tumours such as adenocarcinoma, myelodysplastic syndromes, and systemic mastocytosis. Clonal eosinophilia is associated with myeloid or lymphoid neoplasms harboring *PDGFRA*, *PDGFRB*, or *FGFR1* rearrangements, Philadelphia-negative myeloproliferative neoplasms, acute myeloid leukaemia, and B- or T-lymphoblastic leukemia/lymphoma featuring marked eosinophilia. Chronic eosinophilic leukemia not otherwise specified (CEL-NOS) is characterized by an AEC exceeding 1,500/µL, blast counts below 20%, increased blasts (>2% in blood or >5% in marrow), and demonstration of clonal cytogenetic or molecular abnormalities in the absence of Philadelphia-positive or negative MPN/MDS overlap and absence of *PDGFRA*/*PDGFRB*/*FGFR1* rearrangements. Idiopathic hypereosinophilic syndrome is diagnosed when the AEC persists above 1,500/µL for more than six months with evidence of organ damage and after exclusion of reactive causes, CEL-NOS, and WHO-defined myeloid neoplasms with eosinophilia [9,10].

In our case, the clues supporting reactive eosinophilia include clinical temporal relationship, lack of organomegaly, falling AEC without therapy, and normal bone marrow blast profile. The association of severe eosinophilia and hypersensitivity reaction with hematogone hyperplasia has not previously been described. It is possible that bone marrow regenerative response, combined with cytokine-driven inflammation, contributed to hematogone expansion.

This case highlights that severe eosinophilia in pediatric patients may closely simulate the clinical and hematological presentation of acute leukemia, necessitating meticulous diagnostic evaluation. When peripheral smears demonstrate blasts or atypical lymphocytes, bone marrow examination together with multiparametric flow cytometry becomes indispensable for accurate lineage assignment and for distinguishing reactive processes from malignant hematopoiesis. Hematogone hyperplasia, a benign regenerative expansion of B-cell precursors, may phenotypically resemble leukemic blasts, requiring expert interpretation to prevent misclassification. Careful clinicopathological correlation is essential to avoid unwarranted invasive procedures or cytotoxic therapy, and prompt recognition of drug-induced eosinophilia is critical to prevent re-exposure to the offending agent and associated morbidity.

## Conclusions

This report presents a rare case of drug-induced severe eosinophilia accompanied by hematogone hyperplasia, initially mimicking acute leukemia. It underscores the diagnostic complexity associated with severe eosinophilia and highlights the value of detailed clinical assessment, bone marrow evaluation, and flow cytometry in distinguishing benign regenerative responses from malignant pathology. Increased awareness of such presentations can prevent overtreatment and reduce psychological burden for families. Early identification and management of reversible causes remain fundamental to optimal pediatric care.
